# A sorghum seed variety identification method based on image–hyperspectral fusion and an improved deep residual convolutional network

**DOI:** 10.3389/fpls.2025.1632698

**Published:** 2025-08-22

**Authors:** Xu Yang, Yihan Chen, Shaozhong Song, Zhimin Zhang

**Affiliations:** ^1^ School of Electronic Information Engineering, Changchun University of Science and Technology, Changchun, China; ^2^ School of Data Science and Artificial Intelligence, Jilin Engineering Normal University, Changchun, China; ^3^ Jilin Academy of Agricultural Sciences Peanut Institute, Gongzhuling, Jilin, China

**Keywords:** artificial intelligence, sorghum seed, variety identification, multi-modal fusion, ResNet models

## Abstract

**Introduction:**

Sorghum is an important food and feed crop. Identifying sorghum seed varieties is crucial for ensuring seed quality, improving planting efficiency, and promoting sustainable agricultural development.

**Methods:**

This study proposes a high-precision classification method based on the fusion of RGB images and hyperspectral data, using an improved deep residual convolutional neural network. A spectrogram fusion dataset containing 12,800 seeds from eight sorghum varieties was constructed. The network was enhanced by integrating depthwise separable convolution (DSC) and the Convolutional Block Attention Module (CBAM) into the ResNet50 framework.

**Results:**

The CBAM-ResNet50-DSC model demonstrated outstanding performance, achieving a classification accuracy of 94.84%, specificity of 99.20%, recall of 94.39%, precision of 94.52%, and an F1-score of 0.9438 on the fusion dataset.

**Discussion:**

These results confirm that the proposed model can accurately and non-destructively classify sorghum seed varieties. The method offers a dependable and efficient approach for seed screening and has practical value in agricultural applications.

## 1 Introduction

Sorghum is a drought and heat-tolerant cereal crop widely used for food, feed, and brewing. Its gluten-free nature and associated health benefits have also drawn increasing attention in developing functional and health-oriented food products (Khoddami et al., 2023). Seed purity refers to the consistency of sorghum seeds in maintaining their characteristic traits, which directly impacts the crop’s yield and quality. During harvesting and storage, impurities may be unintentionally mixed into seed lots, leading to economic losses in agricultural production and processing. Moreover, some individuals or companies may intentionally substitute inferior sorghum seeds for high-quality varieties in the seed market to gain additional profit (Zhang et al., 2017). Therefore, developing a rapid and nondestructive detection technique to screen and grade sorghum seeds before they enter the market is essential, ensuring effective agricultural production implementation, quality control, and market supervision.

Traditional methods for seed identification include visual inspection (Shahin et al., 2006), flotation (Torchio et al., 2014), microscopic analysis (Asmussen et al., 2015), chemical testing (Chen et al., 2016), and germination experiments (Andersson et al., 2007). Although these approaches are simple and easy to implement, they are time-consuming and highly subjective, making them insufficient to meet the demands of modern agriculture. Therefore, there is an urgent need for a rapid, accurate, and nondestructive method for identifying and classifying sorghum seeds.

In recent years, image processing and deep learning approaches have received a lot of interest in the subject of seed classification. For example, Franco C showed that by combining data augmentation with deep convolutional neural networks, seed vigor could be predicted with up to 90% accuracy using simple RGB information, such as shape, color, and size (Franco et al., 2020). Similarly, Masuda K used deep learning and interpretable AI approaches to perform noninvasive diagnosis of seedless/seeded internal features in persimmon fruits, with a VGG16 model classification accuracy of up to 89% from simple RGB photos (Masuda et al., 2021). In another study, Sunil, G used the VGG16 deep learning classifier to classify RGB images of four weeds (horseshoe grass, kochia, ragweed, and waterhemp) and six crops (black beans, canola, corn, flax, soybeans, and sugar beets). The results showed that the average Fl score of the VGG16 model classifier ranged between 93% and 97.5 (Sunil et al., 2022).

In summary, RGB data collected by industrial cameras, paired with deep learning models, performed well in seed classification. However, relying solely on RGB photos does not completely utilize the spectral information inside the seeds, resulting in certain limitations in categorization accuracy. As a result, hyperspectral imaging technology is a cutting-edge technology that has advanced rapidly in recent years, and it has been integrated with artificial intelligence algorithms to create a new nondestructive detection technique (Kamruzzaman et al., 2016). For example, Soares and SFC employed near-infrared hyperspectral imaging (NIR-HSI) to quickly and non-destructively classify cotton seed varieties. The NIR-HSI, conventional NIR, and conventional VIS-NIR datasets were correctly classified at 98.0%, 89.7%, and 91.7%, respectively, using partial least squares discriminant analysis (Soares et al., 2016). Similarly, An, JL introduced a unique feature extraction method called Low-Rank Tensor Approximation (LRTA) based on hyperspectral images, which improved accuracy by 4% over the old method (An et al., 2023). In another study, Malik showed that integrating hyperspectral imaging (HSI) and convolutional neural networks (CNNs) could quickly and non-destructively estimate the tofu quality of soybean seeds with 96-99% accuracy (Malik et al., 2024). Recently, Yao et al. proposed Spectral Mamba, an efficient state-space model for hyperspectral image classification (Yao et al., 2024), while Pang et al. introduced SPECIAL, a CLIP-based zero-shot classification framework that eliminates the need for manual annotations (Pang et al., 2025), providing new directions for efficient and generalizable HSI analysis.

In recent years, to enhance feature extraction capability and computational efficiency in agricultural image analysis, attention mechanisms (such as SE, ECA, and CBAM) and depthwise separable convolutions (DSC) have been widely introduced into various detection and classification tasks. Jiang et al. proposed a deep learning-based method for dense Muscovy duck detection. By integrating CBAM modules into the YOLOv7 framework, they developed the CBAM-YOLOv7 model. Experimental results demonstrated that this method outperformed SE-YOLOv7 and ECA-YOLOv7 in terms of accuracy, recall, and mAP, confirming the effectiveness of attention mechanisms in dense livestock detection tasks (Jiang et al., 2022). Guo et al. proposed an improved SSD-based method for cotton leaf disease detection to address the problems of large model size and low detection accuracy. By introducing the lightweight MobileNetV2 as the backbone and integrating SE, ECA, and CBAM attention mechanisms, the model significantly reduced parameters and computation while enhancing detection speed and accuracy. Among the variants, the SSD_MobileNetV2+ECA model achieved the highest precision, recall, F1-score, mAP, and FPS, demonstrating that attention mechanisms can effectively enhance feature representation and improve detection performance under complex conditions (Guo et al., 2024). Tyagi et al. proposed a hyperspectral imaging approach combined with improved depthwise separable convolution to assess fruit maturity. Applied to kiwifruit and avocado, the model achieved higher accuracy in predicting maturity, firmness, and sugar content, outperforming state-of-the-art methods (Tyagi et al., 2024). In summary, although existing studies have made progress in seed classification using RGB images and hyperspectral techniques, and methods such as attention mechanisms and depthwise separable convolutions have shown potential in enhancing model performance and efficiency, each approach has its limitations. RGB-based methods lack internal spectral information, while hyperspectral approaches often face high data complexity. Moreover, multi-modal studies specifically targeting sorghum seeds remain limited, highlighting the need for further research to improve classification accuracy and practical applicability. As a result, our project team has previously investigated the merging of geometric and textural features taken from photos with hyperspectral data to create multi-modal feature vectors and categorize them using machine learning algorithms. The results demonstrate that multi-modal data fusion can significantly increase classification performance (Bi et al., 2024). However, in real applications, the preprocessing procedure of integrating multi-modal data into one-dimensional vectors is complex, increasing the preparation workload. As a result, this paper presents a novel multi-modal fusion technique and employs an upgraded ResNet network model for classification.

The main contributions and novelties of this work are summarized as follows:

1. We propose a novel data-level multi-modal fusion strategy that transforms one-dimensional hyperspectral data into two-dimensional reflectance curve images and concatenates them with RGB images to form a unified spectrogram-like input. This early-stage data fusion preserves both spatial and spectral characteristics in a structurally consistent format (224*224*3), enabling the network to extract complementary information more effectively and facilitating end-to-end learning.
2. We design an enhanced ResNet50-based classification model by integrating the Convolutional Block Attention Module (CBAM), which strengthens the model’s ability to focus on critical spatial and channel features, leading to improved feature discrimination and classification performance.
3. To further reduce model complexity and improve computational efficiency, we incorporate depthwise separable convolution (DSC) into the network. This not only lowers the number of parameters and FLOPs but also maintains high accuracy, making the model more suitable for large-scale or resource-constrained applications.

Overall, this study introduces a lightweight and effective framework for high-resolution sorghum seed classification, demonstrating the value of data-level fusion in enhancing feature representation and model performance.

The following of this article was organized as the section “Materials and Methods” described the details of the datasets and the overview of the methods, the experimental results were described and discussed in the section “Results and Discussions,” and the section “Conclusions” was the concluding remarks.

## 2 Materials and methods

### 2.1 Image acquisition and preprocessing

#### 2.1.1 Data source and acquisition

The Jilin Academy of Agricultural Sciences, Jilin Province, supplied eight distinct sorghum seed varieties, including JZ127, JZ136, JZ141, JZ159, JZ160, JZ177, JZ186, and JZ187, which were employed in this experiment. There were 12,800 seeds in total, 1600 seeds in each type. Training, validation, and testing were the three groups into which the dataset was split in a 7:2:1 ratio.

#### 2.1.2 Data acquisition and preprocessing

##### 2.1.2.1 RGB image data

**Figure 1:**
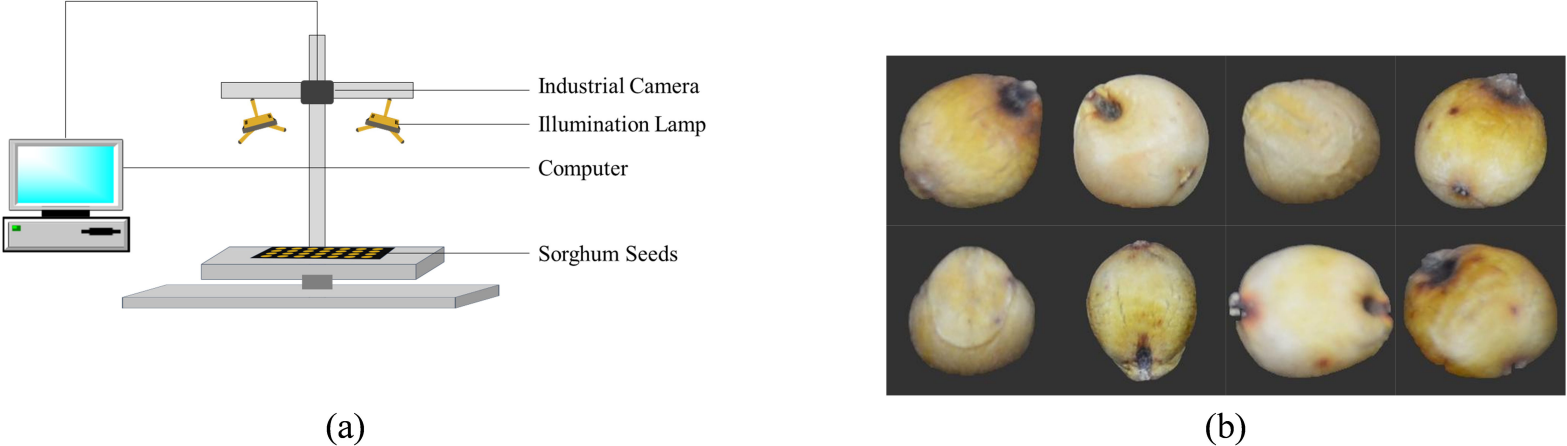
**(a)** Schematic diagram of the RGB imaging setup for sorghum seeds **(b)** RGB images of eight sorghum seed varieties.

A Nikon camera (Nikon D7100) was used to take RGB pictures of these seeds, and **Figure 1a** displays the RGB capture scheme. The seeds were carefully chosen and validated by professionals before images were taken to guarantee that the samples were entire, consistently shaped, and free of dust and contaminants. Every seed that was chosen acted normally looked tidy, and showed no signs of damage. For imaging, 1600 samples of each type were randomly picked; the sample size was selected to accommodate the substantial data needed for the deep learning model (Wen, 2020). **Figure 1b** displays the RGB pictures of the gathered sorghum seeds.

**Figure 2:**
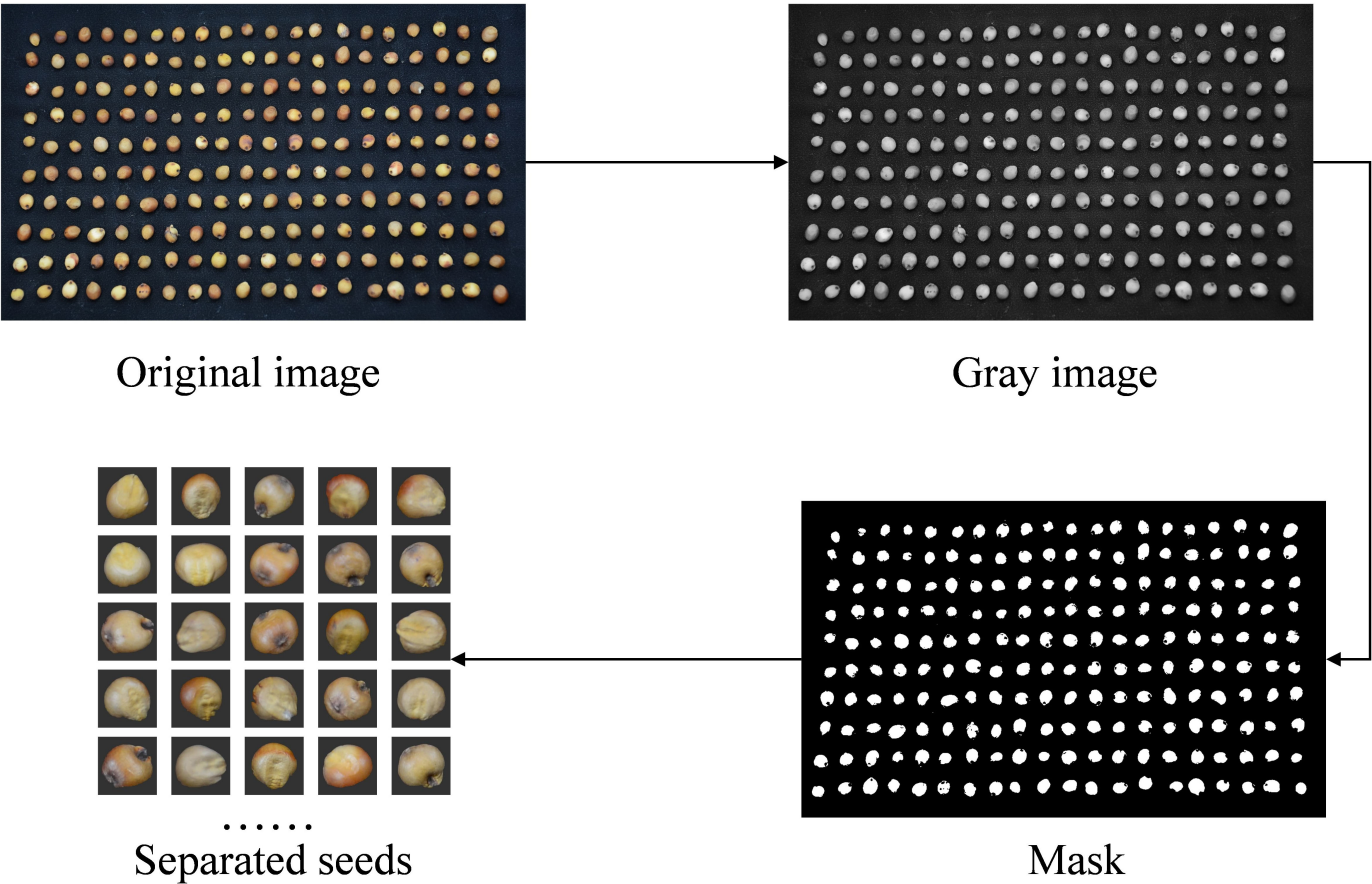
Sorghum seed image cutting preprocessing.

The main goal of sorghum variety identification is to ensure sorghum seeds are pure, particularly to confirm the legitimacy of individual seeds. An image with several seeds must be segmented to employ a single seed recognition technique to differentiate between various sorghum seed types. The original image is first transformed to greyscale to highlight brightness-related elements and exclude color information. A binarized image is then produced by applying automatic global thresholding and morphological filtering procedures, simplifying the image and extracting the target contours. Lastly, morphological filtering was used to score and mask the sorghum seed area in the binarized image. After that, it was divided into separate 224*224 sorghum seeds, yielding 12,800 raw photos. **Figure 2** depicts the picture-cutting procedure.

##### 2.1.2.2 Hyperspectral data

**Figure 3:**
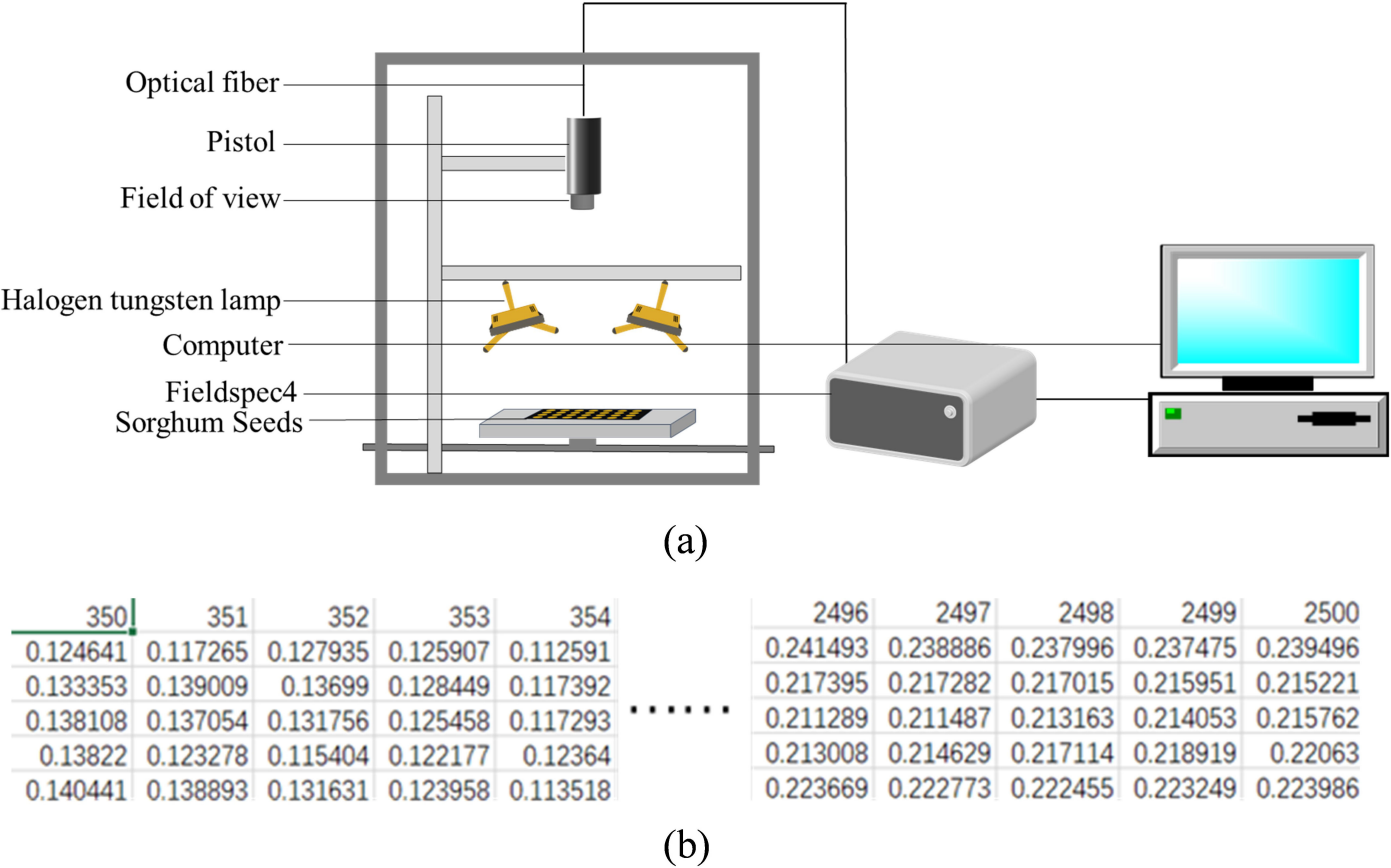
**(a)** Schematic diagram of the hyperspectral data acquisition setup for sorghum seeds **(b)** Schematic representation of the raw spectral data storage format for sorghum seeds.

The spectral data of sorghum seeds was obtained in this experiment using a FieldSpec4 ground spectrometer; **Figure 3a** displays the schematic diagram of the bright light data acquisition. The instrument operates in the visible (VNIR), near-infrared (NIR), and short-wave infrared (SWIR) bands, which span the wavelength range of 350–2500 nm. It is ideal for fine spectrum analysis due to its broad wavelength range, high signal-to-noise ratio (SNR up to 9000:1), and excellent spectral resolution (VNIR 1–3 nm, NIR 3–5 nm, SWIR 5–10 nm). In this experiment, the spectral characteristics of sorghum seeds may be efficiently characterized for seed classification utilizing hyperspectral data obtained with the FieldSpec4 ground spectrometer. Because of its structure and effectiveness, a one-dimensional form is typically employed to store spectral data. **Figure 3b** displays the one-dimensional spectrum data.

##### 2.1.2.3 2D fusion of image data with hyperspectral data

**Figure 4:**
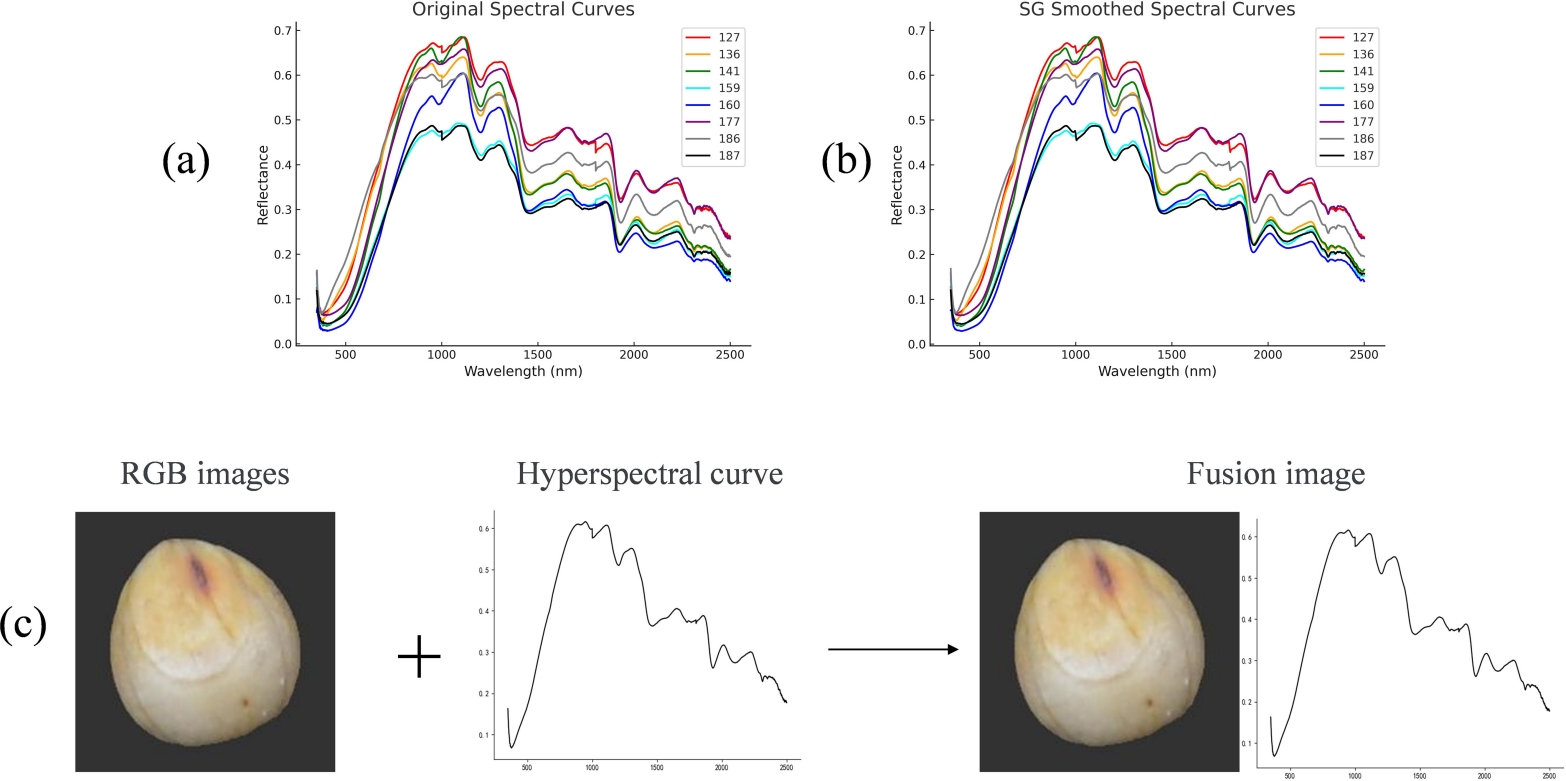
**(a)** Raw hyperspectral curves **(b)** Preprocessed hyperspectral curves **(c)** Fusion of RGB data and hyperspectral data.

In earlier studies, this project team successfully built multi-modal feature vectors, applied machine learning techniques for classification, and merged geometric and textural aspects of images with hyperspectral data. The findings demonstrate that the classification performance is much enhanced by multi-modal data fusion. The multi-modal data must be combined into one-dimensional vectors using this fusion approach, and the preprocessing step is challenging, adding to the data processing workload. This paper suggests a novel approach to data fusion: upscaling one-dimensional spectral data to two-dimensional curves and fusing them with two-dimensional picture data. This approach increases the fusion process’s efficiency while streamlining the data preprocessing step. In particular, wavelengths and their related reflectance values are employed to record the spectral data, with the wavelength serving as the horizontal coordinate and the reflectance as the vertical coordinate. The one-dimensional spectral data are plotted intuitively as spectral reflectance curves using data visualization tools (such as Python’s matplotlib library). Using Python visualization tools such as matplotlib, the processed spectral data are plotted into intuitive reflectance curves, clearly reflecting the sample’s spectral response across different wavelengths and providing a solid foundation for further data analysis and fusion. **Figure 4a** illustrates a typical spectral curve of collected sorghum seeds. To enhance curve smoothness and readability, Savitzky-Golay (SG) filtering is applied prior to plotting, effectively suppressing high-frequency noise while preserving key structural features, as shown in **Figure 4b**. Given that this study fuses spectral and RGB image data in image format for classification, more complex preprocessing techniques such as SNV or MSC normalization-which may distort curve shapes or introduce redundancy were deliberately avoided. Instead, SG filtering is selected as the sole preprocessing method due to its simplicity, low computational cost, and excellent shape-preserving capability, ensuring the physical and visual integrity of the spectral data for image fusion and model input.

Subsequently, a new two-dimensional spectral fusion dataset was constructed by horizontally concatenating the RGB images with the preprocessed spectral curve images. As shown in **Figure 4c**, the original RGB image with size 224*224*3 and the grayscale spectral curve image with size 224*224*1 were first aligned in format. To address the channel mismatch, the grayscale image was replicated across the R, G, and B channels to form a pseudo-color image with size 224*224*3, maintaining the visual appearance while ensuring structural compatibility. The two images were then stitched side by side to form a unified fusion image with a final resolution of 224*448*3 This fusion strategy preserves the intuitive visual information of the RGB image while incorporating the key spectral features from the hyperspectral data. It ensures structural consistency in the fused input and enhances the expressive power of the data, providing a richer and more integrated multi-modal representation for deep learning-based classification.

### 2.2 Building the model

Gradient degradation and gradient vanishing issues frequently arise during the training phase of convolutional neural networks (CNNs) as their depth grows, impacting the model’s convergence rate and ultimate accuracy (Wenchao and Zhi, 2022). Although the gradient vanishing and the ResNet family of networks somewhat mitigate explosion issues in deep neural networks, performance deterioration may still occur during deep model training (Traore et al., 2018). This paper introduces the attention mechanism into the network structure to improve sorghum seeds’ recognition performance and classification accuracy. It replaces some standard convolutions with depth-separable convolutions to enhance the model’s ability to extract key information. In order to create a fast and nondestructive variety classification method based on sorghum seed image data and hyperspectral data, this experiment will use deep learning algorithms for eight different types of sorghum seeds (ResNet18, ResNet34, ResNet50, ResNet101, SENet-ResNet50, CBAM-ResNet50, ECA-ResNet50, CBAM-ResNet50-DSC, eight residual network models).

#### 2.2.1 ResNet model

**Figure 5:**
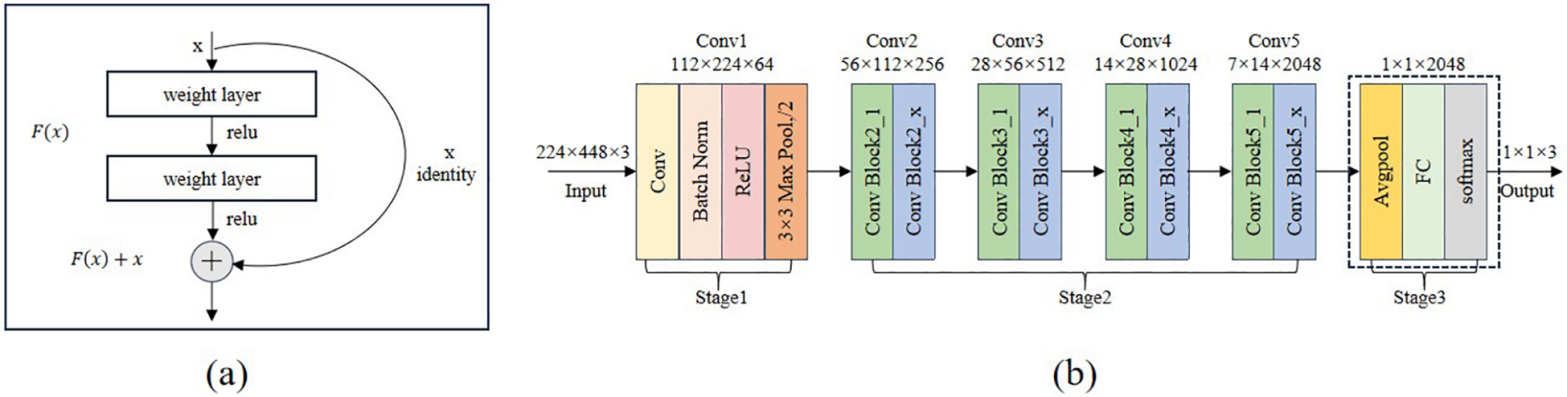
Structure of the residual block and structure of the Resnet50 network. **(a)** the residual block **(b)** ResNet50 structure diagram.

(He et al., 2016) proposed the deep neural network structure known as ResNet. While increasing the network layers, this network successfully addresses the gradient vanishing issue and enhances parameter consumption efficiency by implementing the residual connection mechanism. ResNet is a popular model choice for sorghum seed detection tasks because of its strong learning ability for complicated features and rapid inference speed, which allows it to perform better while maintaining good generalization performance. The Residual Block, the fundamental unit structure of the residual network, is seen in **Figure 5a**. The structure uses a Skip Connection and a primary path to implement feature learning and transfer. By applying two convolutional transforms to the input feature x and utilizing the ReLU activation function following each convolution, the main path determines the residual F(x). The input feature x is then sent straight to the output by the bypass connection, where it is combined with the residuals F(x) that the primary path has learned to create the final output F(x)+x. This design successfully increases the network’s performance capability and training efficiency by preserving the information of the input features and resolving the gradient vanishing issue in the deep network. The residual block is the fundamental building element of ResNet, which introduces skip connections and identity mapping to address the gradient vanishing and gradient explosion issues in deep neural networks. **Figure 5b** illustrates the overall architecture of the residual network using ResNet50 as an example. This network adopts a fully convolutional structure and does not include any fully connected layers that enforce fixed input dimensions during the feature extraction stage, thereby exhibiting strong adaptability to varying input sizes. As long as the input image maintains valid spatial dimensions after multiple convolution and pooling operations, the network can operate stably with consistent output structure. Accordingly, this study uses a concatenated input image with dimensions of 224*224*3, which can be directly fed into the ResNet50 model for feature extraction and classification without any structural modifications. In the first stage, a 7*7 convolutional layer followed by a 3*3 max pooling layer downsamples the input to reduce its spatial resolution. Then, in the second stage, the residual modules Conv2, Conv3, Conv4, and Conv5 are introduced sequentially to extract higher-level semantic features. Throughout this process, the spatial dimensions of the feature maps gradually decrease from 56 to 7 in height and from 112 to 14 in width, while the number of channels increases from 256 to 2048. In the third stage, global average pooling compresses the feature map to 1*1*2048, and the final classification result is obtained through a fully connected layer.

#### 2.2.2 Attention mechanisms

##### 2.2.2.1 Squeeze-and-excitation networks

SENet (Squeeze-and-Excitation Networks) is a deep learning model based on the attention mechanism, which dynamically models the channel relationship of feature maps by introducing the Squeeze-and-Excitation (SE) module to enhancing the attention to the essential features and suppressing the irrelevant features (Li et al., 2020). The input feature maps are constantly adjusted by SENet’s Squeeze-and-Excitation module in three stages: Squeeze, Excitation, and Reweighting. The global description of each channel is first obtained in the Squeeze stage by using Global Average Pooling (GAP) to compress the input feature map H×W×C in the spatial dimension. In particular, each channel’s global feature $z_{c}$ is calculated using Equation 1.


(1) $$\begin{array}{c} z_{c}=\frac{1}{H\times W}\sum_{i=1}^{H}\sum_{j=1}^{H}X_{c}\left(i,j\right) \end{array}$$

A vector with dimensions of 1×1×C is the end product.

A two-layer Fully Connected Network (FC) captures the nonlinear interaction between channels and generates dynamic channel weights in the Excitation step. The nonlinear changes are introduced using the ReLU activation function after the first layer of the fully linked network decreases the number of channels to r times the initial number (often r = 16, or dimensionality reduction). The second layer of the completely linked network then uses the Sigmoid activation function to create the normalized weight s, which has a size of 1×1×C and returns the number of channels to its initial size.

The final output feature map is created by multiplying the generated channel weights by the input feature map channel by the channel during the Reweighting stage. In particular, the output feature $X^{c}$ is calculated as shown in Equation 2:


(2) $$\begin{array}{c} X^{c}=s_{c}\cdot Xc \end{array}$$

**Figure 6:**
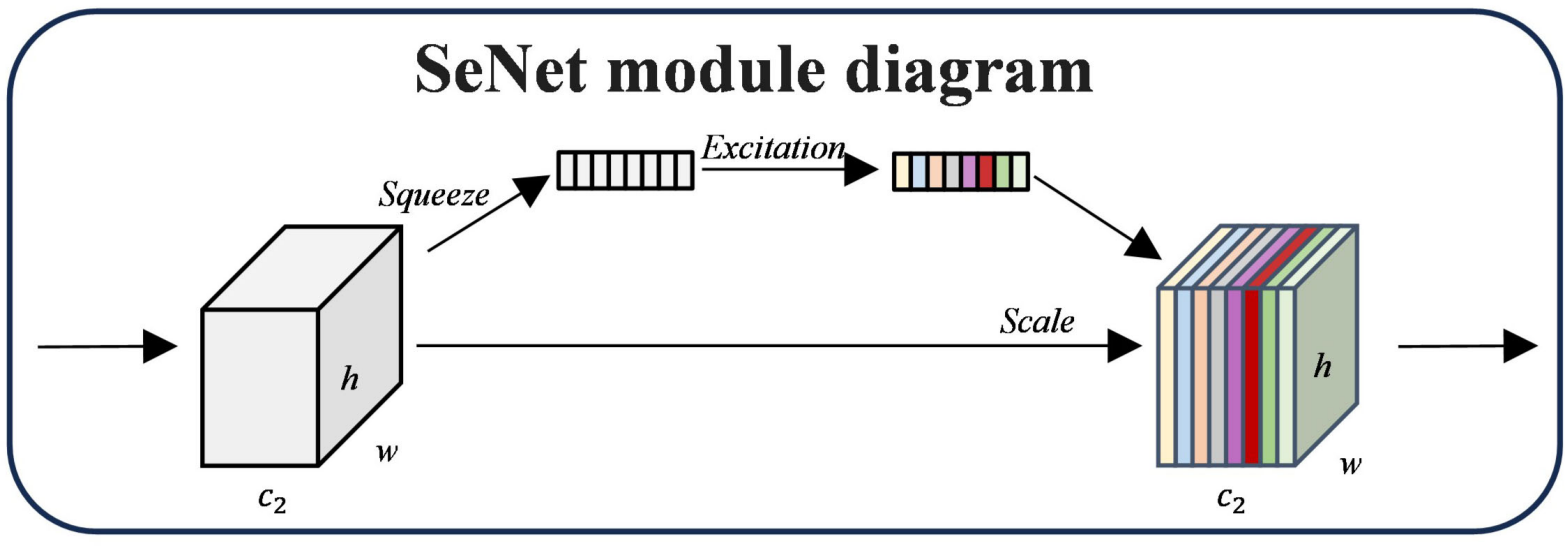
SE module diagram.

Where the weight of channel c is denoted by $s_{c}$ . The SE module can enhance the expressiveness and classification performance of the model by emphasizing significant features and suppressing unimportant ones through a dynamic weighting technique. **Figure 6** displays the SE module diagram.

##### 2.2.2.2 Convolutional block attention module

**Figure 7:**
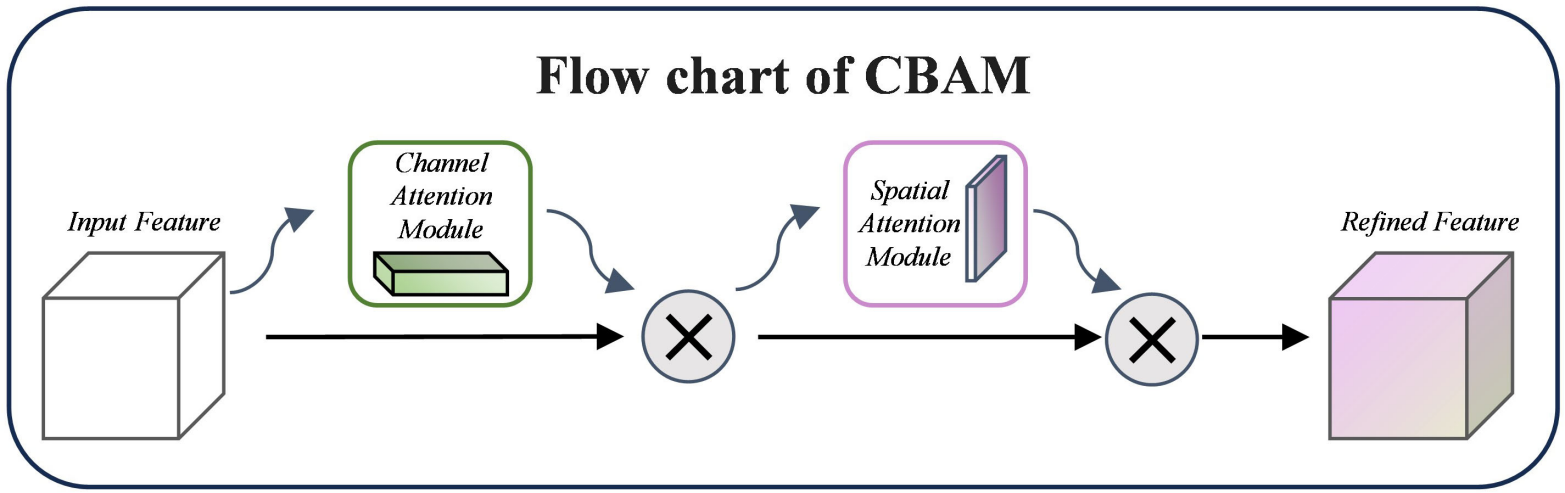
CBAM module diagram.

CBAM (Convolutional Block Attention Module) is a lightweight and efficient attention mechanism that can significantly improve the performance of Convolutional Neural Networks (Woo et al., 2018). Applying channel and spatial attention to the input feature maps highlights significant channels and crucial spatial locations. While the Spatial Attention module creates spatial weights using pooling and convolution operations, the Channel Attention module uses global average pooling and maximum pooling to extract global context information and build channel weights. In **Figure 7**, the CBAM structure is displayed.

The output $\;M_{C}\;\left(F\right)$ of the channel attention module can be calculated using Equation 3:


(3) $$\begin{array}{c} M_{C}\;\left(F\right)=\sigma \left(MLP\left(AvgPool\left(F\right)\right)+MLP\left(MaxPool\left(F\right)\right)\right) \end{array}$$

MLP stands for multilayer perceptual machine, σ for sigmoid activation function, F for input feature map, and AvgPool and MaxPool for global average pooling and maximum pooling operations, respectively, in Eq.

And the loss $M_{S}\left(F\right)$ of the spatial attention module can be calculated by Equation 4:


(4) $$\begin{array}{c} M_{S}\left(F\right)=\;\sigma \;\left(\;f^{7\times 7}\left(\left[AvgPool\left(F\right);MaxPool\left(F\right)\right]\right)\right) \end{array}$$

[AvgPool(F);MaxPool(F)] indicates sewing together the average pooling and maximum pooling results along the channel axis, whereas $f^{7\times 7}$ indicates a 7×7 convolution operation.

##### 2.2.2.3 Efficient channel attention

**Figure 8:**
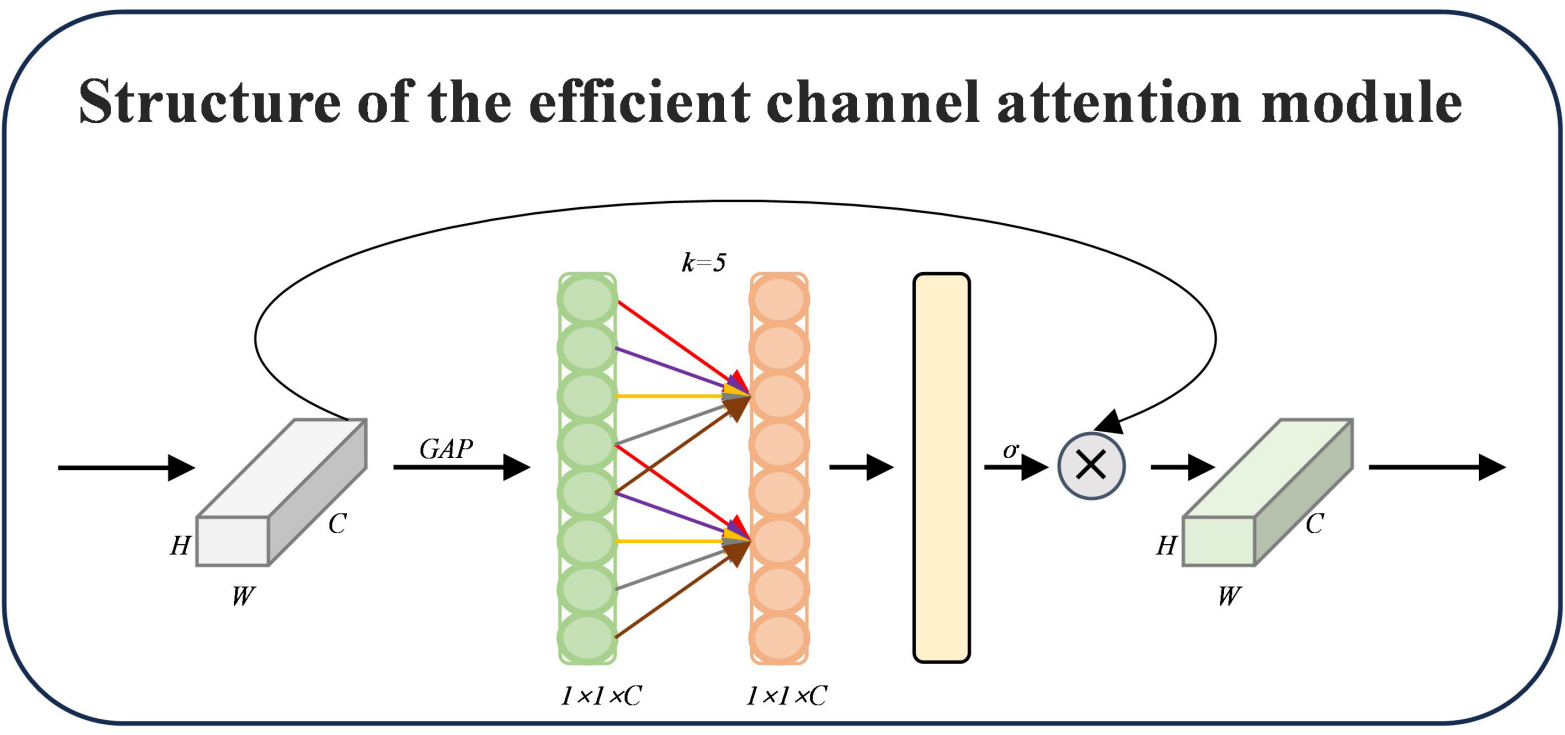
ECA module diagram.


ECA (Efficient Channel Attention) is an effective channel attention method that dramatically lowers the computational complexity by eliminating the fully connected layer from the conventional SE module and utilizing 1D convolution to capture the local interactions between channels (Wang et al., 2019). Using an adjustable convolution kernel size that dynamically interacts with the number of channels, ECA can guarantee that the model is lightweight while significantly enhancing network performance. The construction of ECA is depicted in **Figure 8**, where a channel description vector $F_{gp}\in R^{C}$ is obtained by first undergoing Global Average Pooling (GAP) on the input feature map $F\in R^{C\;\times \;H\;\times \;W\;}$ and then extracting the global semantic information of each channel. Instead of using a fully connected layer, ECA dynamically determines the size k of the one-dimensional convolution kernel based on the number of channels *C*, which is calculated using Equation 5. This allows for the realization of local cross-channel interactions without dimensionality compression and, in the end, generates the channel attention weights. This allows for the efficient modeling of the interrelationships between channels.

(5) $$\begin{array}{c} k=\left[\frac{log_{2}\left(C\right)}{\gamma }\;+\;b\;\right] \end{array}$$

Where the hyperparameters are b and γ, the local interactions between channels are then captured by a 1D convolution operation using k, which produces the channel weights $M_{C}\in R^{C}$ using a Sigmoid activation function. Lastly, the improved feature maps F’ are obtained by multiplying the weights $M_{C}$ by the input feature maps channel by channel.

##### 2.2.2.4 Introduction of attention mechanisms

**Figure 9:**
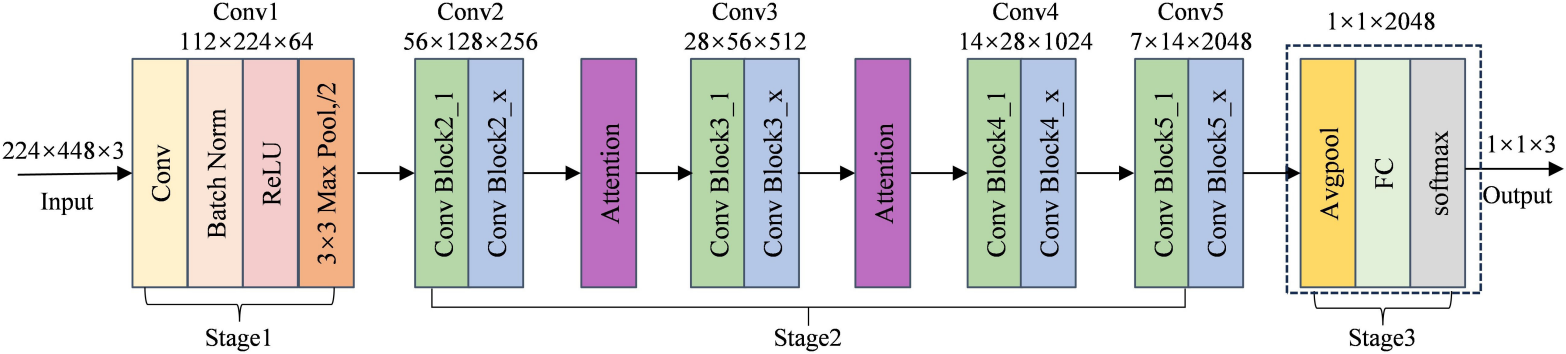
ResNet50 model with integrated attention mechanism.

The architecture of the ResNet50 network improved with attention mechanisms is shown in **Figure 9**. In order to increase its emphasis on significant characteristics and boost classification performance, this model incorporates attention modules at many critical stages compared to the standard ResNet50 framework. The network’s first convolutional layer (Conv1) extracts low-level information. Following Conv2 and Conv3, attention modules are added in the convolutional stages (Stage 2). For comparison study, these modules (designated as *Attention*) stand for SENet, CBAM, and ECA, which are separately included in ResNet50. Lastly, the network uses a fully connected layer (FC), a Softmax layer, and global average pooling (AvgPool) to classify. The residual network’s performance in complex tasks can be improved by better focusing on important feature areas by incorporating attention processes.

#### 2.2.3 Depthwise separable convolution

**Figure 10:**
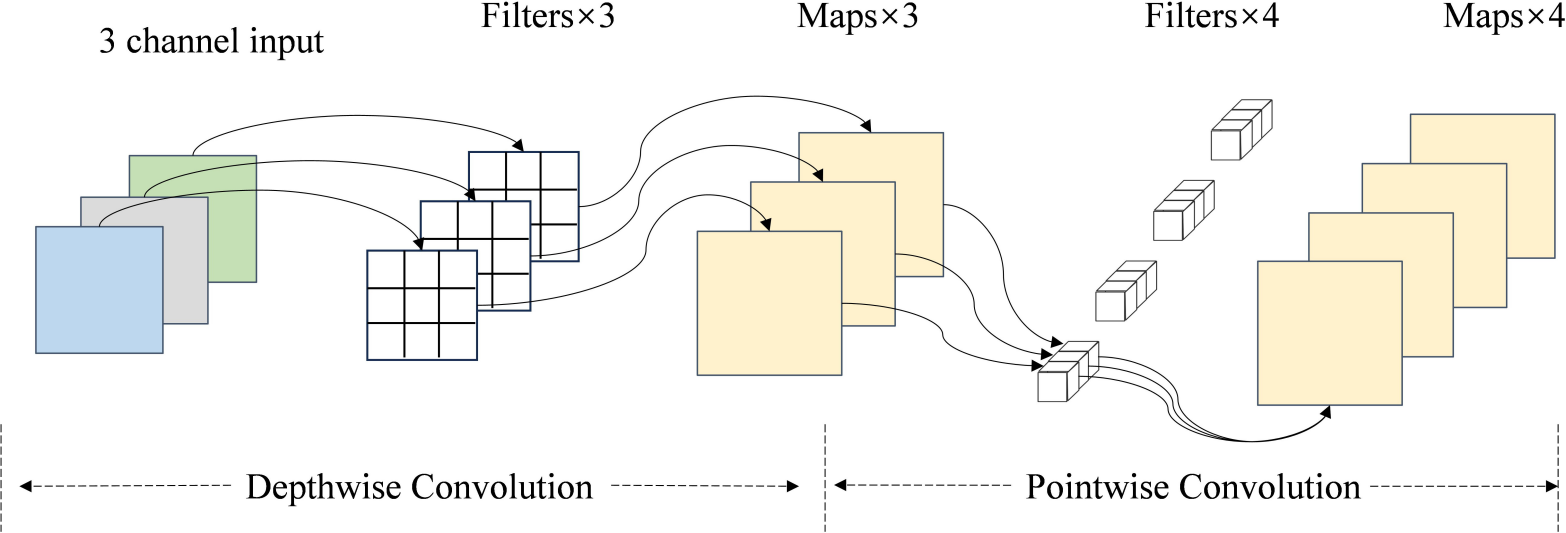
Structure of the depthwise separable convolution.

Additionally, we added depth-separable convolution to the sorghum seed recognition model’s later, residual blocks to lower the network model’s computational expense and time consumption. As illustrated in **Figure 10**, the Depthwise Separable Convolution comprises depth and point-by-point convolution (Chollet, 2017). By breaking down the computational process, depthwise separable convolution drastically lowers the computational cost and parameter count compared to traditional convolution. Conventional convolution, which has a high computational cost, combines channel feature fusion and spatial feature extraction into a single operation. Each convolution kernel acts on all input channels to produce an output channel. Deep separable convolution, on the other hand, divides this process into two steps: the first stage uses deep convolution to extract spatial features by performing the convolution operation independently on each channel, and the second stage uses point-by-point convolution in the channel dimension for weighted combination to realize channel feature fusion. This independent design reduces the amount of computation while maintaining the feature extraction capability of the model, which is an essential component of lightweight models.

#### 2.2.4 Proposed model

**Figure 11:**
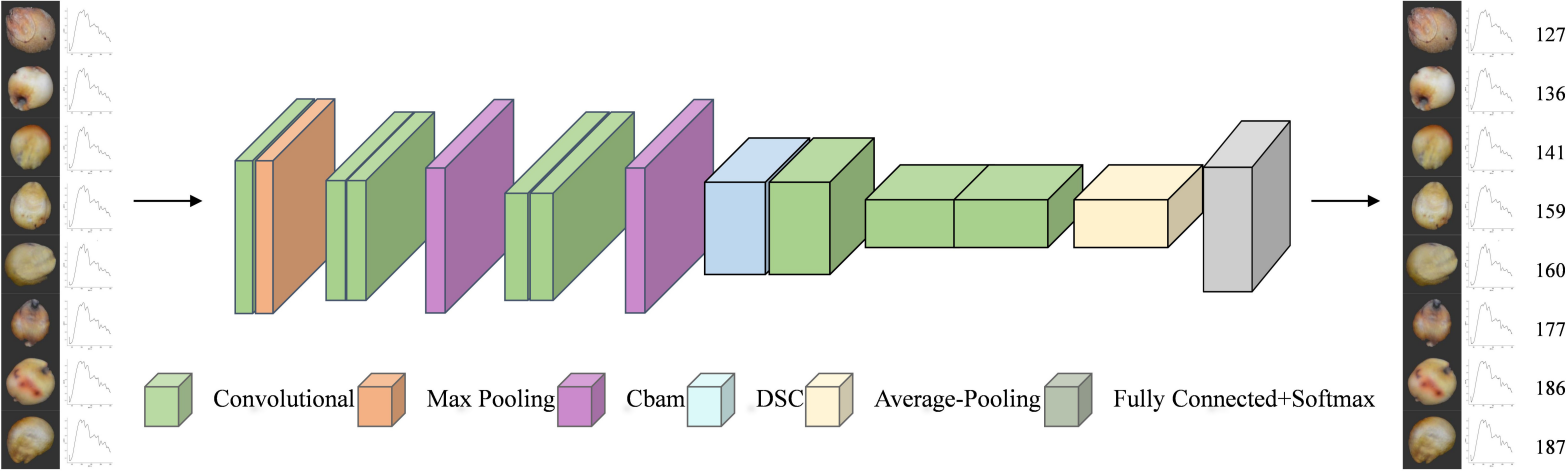
Classification process.

The final process of classifying sorghum seeds by the improved network is shown in **Figure 11**. Depth separable convolution (DSC) at various convolutional layers and the CBAM attention mechanism are introduced in this network design to maximize the model performance. Through the weighting mechanism of channel attention and spatial attention, the CBAM module, which is inserted explicitly after the Conv2 and Conv3 convolutional layers, enhances the model’s capacity to concentrate on essential features and lessens interference from background noise. Furthermore, by breaking down the standard convolution into depth convolution and point-by-point convolution, depth separable convolution (DSC), which is employed in the Conv4 convolutional layer, not only lowers the computational complexity and number of parameters but also enhances computational efficiency while ensuring the feature extraction capability. This strategy greatly optimizes the model’s resource usage while increasing the classification accuracy of sorghum seeds by enabling the network to fuse multi-modal input from RGB images and spectral curves more effectively.

### 2.3 Overall flow chart

**Figure 12:**
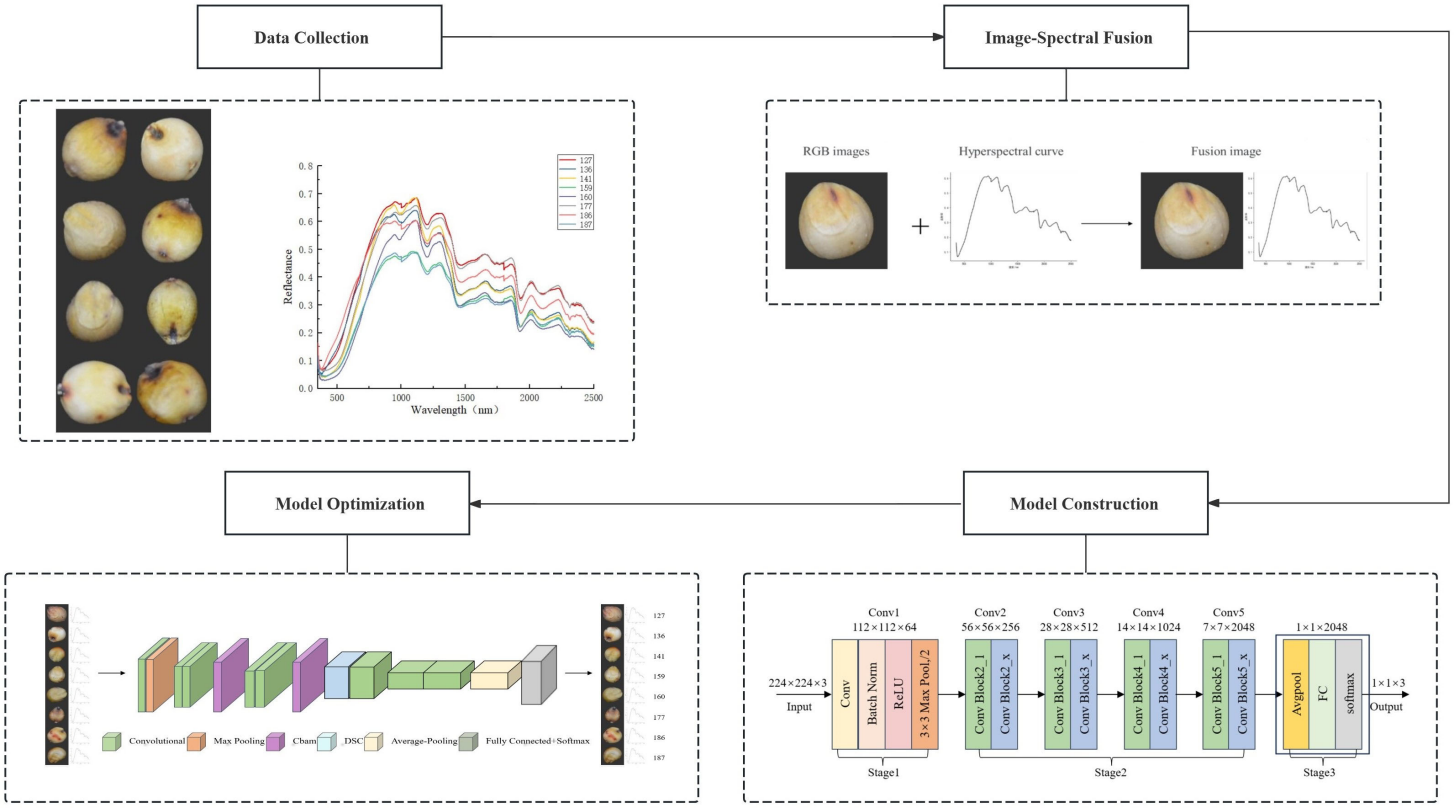
Overall flow chart.

**Figure 12** shows a general flow chart. The entire image processing and model-building procedure for sorghum seeds is depicted in this figure. First, an industrial camera and hyperspectral acquisition equipment gather RGB and hyperspectral data from seeds. The appearance features of the RGB images are extracted using a seed segmentation module, and the spectral features of each pixel are extracted from the hyperspectral data using a spectral curve. In order to increase the accuracy of classification and recognition, the RGB and hyperspectral data are fused to create a feature map that blends spectral and spatial information. An attention mechanism and deep separable convolution were added to the model design using the ResNet residual network to further improve the classification and recognition performance of sorghum seeds. In order to accomplish practical and precise seed recognition, the entire approach focuses on multi-modal data fusion and lightweight model construction.

### 2.4 Indicators for model evaluation

The model’s classification performance was thoroughly evaluated in this work using five widely used assessment metrics: accuracy, specificity, recall, Precision, and F1-score. A comprehensive evaluation of the model across various class distributions is provided by the F1-score, which is the reconciled mean of Precision and Recall. Accuracy, on the other hand, reflects the overall correctness of the model’s classification; Specificity measures the model’s ability to identify negative class samples and emphasizes the importance of reducing false alarms; Recall indicates the model’s sensitivity to positive class samples and focuses on lowering underreporting; and Precision is used to assess the model’s accuracy in predicting positive classes, reflecting the reliability of the classification results. By properly evaluating the model’s strengths and weaknesses locally and overall, combining these metrics enables a thorough examination of the model’s performance in the classification task. It serves as a foundation for additional optimization.

### 2.5 Experimental procedures

The following experimental environment was used for this study: Windows 11 as the operating system, the 12th generation Intel^®^ Core™ i9-12900K (3.20 GHz) as the processor, the NVIDIA GeForce RTX 3090Ti as the graphics configuration, and Pycharm 2021 Community Edition as the integrated development environment. The PyTorch deep learning framework was used to build and train the sorghum seed categorization model. During the model training process, the SGD (Stochastic Gradient Descent) optimizer was selected, with the initial learning rate set to 0.001, and the weight parameters were adjusted to optimize the network loss function. Each epoch represents a complete training cycle for the entire sorghum seed dataset, and its maximum number of rounds was set to 50 in order to obtain the optimal value of the loss function during the training process. In addition, the minimum batch size was set to 8, the momentum parameter was set to 0.9, and the weight decay coefficient was set to 0.01 to enhance the generalization ability of the model and suppress overfitting.

## 3 Results and discussions

### 3.1 Fusion of RGB image data with hyperspectral data

The classification performance of RGB, hyperspectral, and RGB & HSI data on several ResNet models is displayed in **Table 1**. The findings demonstrate that combining RGB and hyperspectral data can significantly enhance classification accuracy. In particular, the ResNet50 model achieves the highest accuracy (0.8961), Precision (0.8969), recall (0.8961), and F1-score (0.8959) with fused data, demonstrating that fused data (RGB & HSI) significantly improves the classification performance in all models. With fused data, other models like ResNet34 also saw notable performance gains. Furthermore, the fused data showed enhanced Specificity and Recall, suggesting that the model could lower the false detection rate and recognize seed classes more accurately by merging RGB and HSI information. Data fusion significantly improved the classification model’s performance by combining the complementary nature of spectral and spatial information. ResNet50 performed best in both data cases, indicating that it can be used as the preferred model for classifying sorghum seeds.

**Table 1:** Comparison of results before and after fusion.

Data	Model	Accuracy	Specificity	Recall	Precision	F1-score
RGB	ResNet18	0.7602	0.9659	0.7607	0.7608	0.7609
	ResNet34	0.8055	0.9722	0.8056	0.8055	0.8054
	ResNet50	0.8664	0.9817	0.8664	0.8662	0.8650
	ResNet101	0.7148	0.9593	0.7148	0.7148	0.7148
HSI	ResNet18	0.7749	0.9679	0.7750	0.7774	0.7748
	ResNet34	0.8438	0.9776	0.8430	0.8441	0.8417
	ResNet50	0.8812	0.9833	0.8827	0.8865	0.8825
	ResNet101	0.7679	0.9682	0.7754	0.7840	0.7752
RGB&HSI	ResNet18	0.8117	0.9758	0.8263	0.8333	0.8260
	ResNet34	0.8656	0.9808	0.8656	0.8657	0.8648
	ResNet50	0.8961	0.9852	0.8961	0.8969	0.8959
	ResNet101	0.8086	0.9727	0.8086	0.8072	0.8072

**Figure 13:**
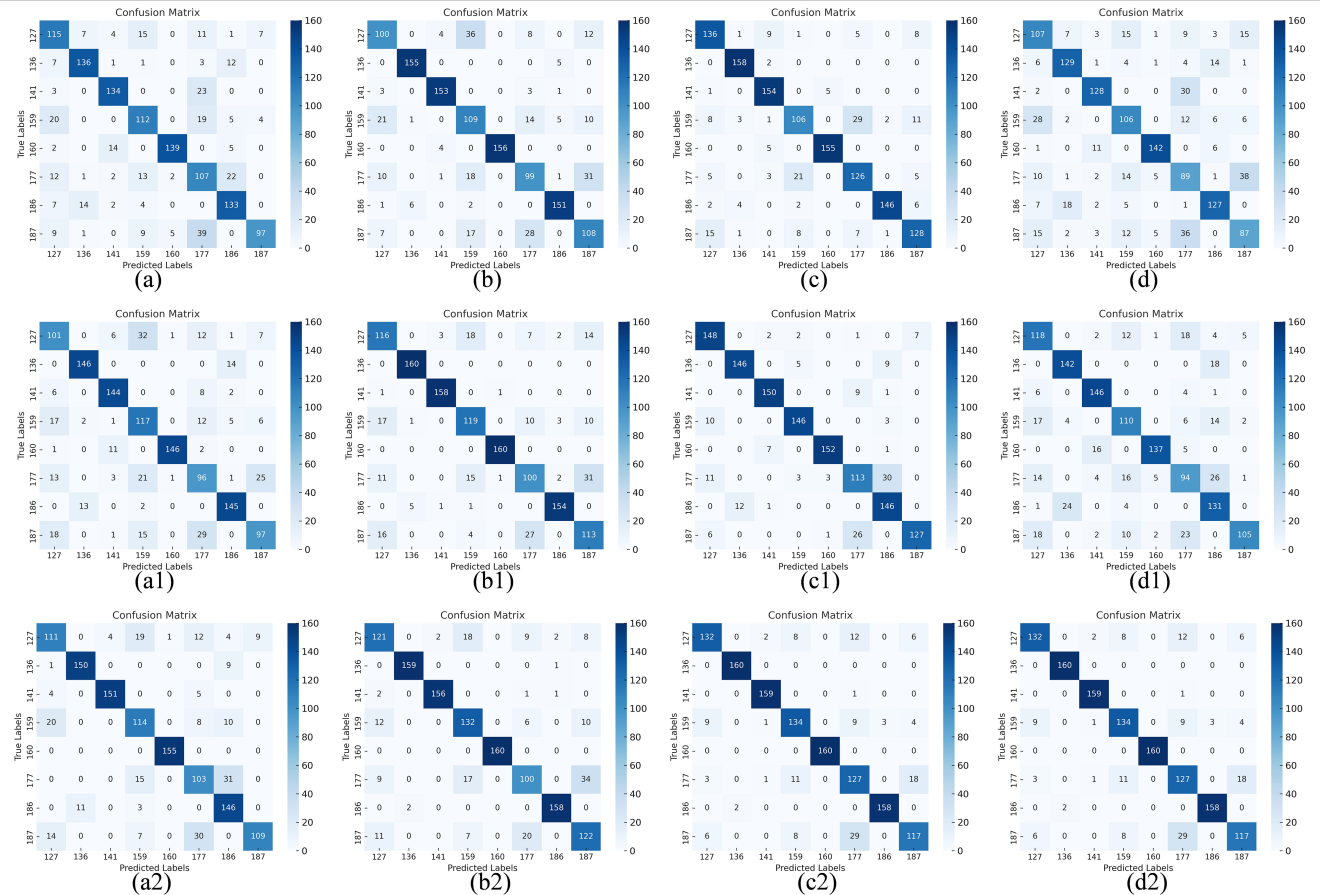
Confusion matrix before and after fusion. Confusion matrix: **(a)** RGB-ResNet18, **(b)** RGB-ResNet34, **(c)** RGB-ResNet50, **(d)** RGB-ResNet101; **(a1)** HSI-ResNet18, **(b1)** HSI-ResNet34, **(c1)** HSI-ResNet50, **(d1)** HSI-ResNet101; (a2) RGB&HSI-ResNet18, (b2) RGB&HSI-ResNet34, **(c2)** RGB&HSI-ResNet50, **(d2)** RGB&HSI-ResNet101.

We compare the visual confusion matrix for the model before and after fusion in **Figure 13** to further confirm that the fusion can enhance the model’s performance. The confusion matrix data describes the sample’s actual categories and the categories that the classifier predicted. Usually, there are four metrics: false positives (FP), false negatives (FN), true positives (TP), and true negatives (TN) (Javanmardi et al., 2021). The fused models (a2-d2) demonstrated a significant increase in the number of correct classifications on the diagonal and a substantial decrease in misclassifications compared to the unfused models (a-d, a1-d1). This suggests that fusing multi-modal data can improve the models’ feature differentiation ability.

### 3.2 Introduction of an attention mechanism

Three distinct attentional processes are added to the ResNet50 network independently for comparison in order to determine the best network model because of its high performance. After combining RGB and HSI data, **Table 2** shows how adding various attention methods (SE, CBAM, and ECA) affects the ResNet50 model’s classification performance. Adding the attention mechanisms enhances the model’s overall classification performance, with CBAM-ResNet50 exhibiting the best results. With an accuracy of 93.20%, recall and Precision of 92.63% and 92.71%, respectively, and an F1-score of 92.43%, CBAM-ResNet50 specifically outperforms the others in every category. While the performance of the residual network with the SE module added is marginally worse than that of the CBAM and ECA modules, it is still far better than the model without the attention mechanism included.

**Table 2:** Results of RGB&HSI data after introducing the attention mechanism.

Data	Model	Accuracy	Specificity	Recall	Precision	F1-score
RGB&HSI	SE-ResNet50	0.9125	0.9886	0.9192	0.9193	0.9190
	CBAM-ResNet50	0.9320	0.9890	0.9263	0.9271	0.9243
	ECA-ResNet50	0.9203	0.9885	0.9199	0.9238	0.9197

Adding the attention mechanism can significantly increase the model’s capacity to identify important features and enhance classification performance, particularly following the fusing of multi-modal data (RGB & HSI). In this experiment, the combination with the best classification effect is CBAM-ResNet50.

**Figure 14:**
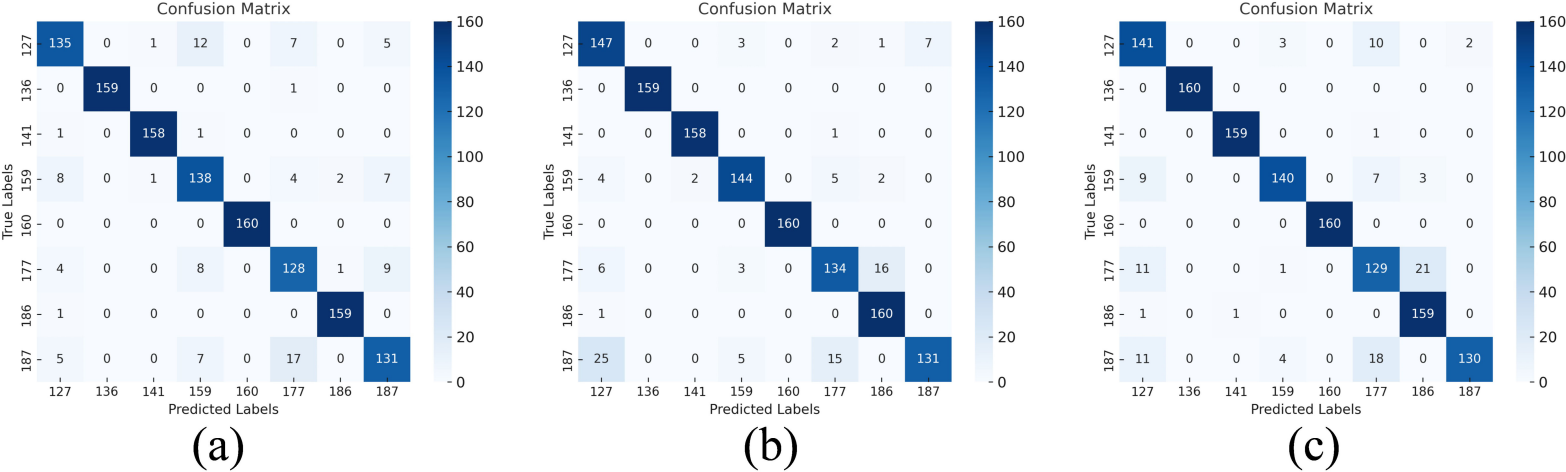
Confusion matrix of the result of introducing the attention mechanism after fusion Confusion matrix: **(a)** RGB&HSI-SE-ResNet50, **(b)** RGB&HSI-CBAM-ResNet50, **(c)** RGB&HSI-ECA-ResNet50.

The confusion matrix in **Figure 14** also shows that adding various attention strategies enhances the model’s classification performance. The mechanism can effectively focus on the important information in the spatial dimension to improve accuracy, as evidenced by the Resnet50 model with the addition of CBAM having the clearest diagonal of the confusion matrix and a further decrease in misclassifications.

### 3.3 Introducing depth separable convolution

The comparison of classification performance outcomes following the addition of depth separable convolution (DSC) to the RGB & HSI-CBAM-ResNet50 models is shown in **Table 3**. The model combines the optimal design of CBAM and DSC based on merging RGB and HSI data to produce good performance in all measures. In particular, the model’s accuracy of 94.84% indicates a high level of classification precision overall; its specificity of 99.20% shows that it can effectively lower the false detection rate; and its recall and precision of 94.39% and 94.52%, respectively, show that it can identify every category in real classification. The capacity of actual classification to identify each category is more balanced. Furthermore, the model’s outstanding performance in striking a balance between recall and Precision is further confirmed by the F1-score, which reaches 94.38%.

**Table 3:** Comparison of introducing depth-separable convolution.

Data	Model	Accuracy	Specificity	Recall	Precision	F1-score
RGB&HSI	CBAM-ResNet50	0.9320	0.9890	0.9263	0.9271	0.9243
RGB&HSI	CBAM-ResNet50-DSC	0.9484	0.9920	0.9439	0.9452	0.9438

**Figure 15:**
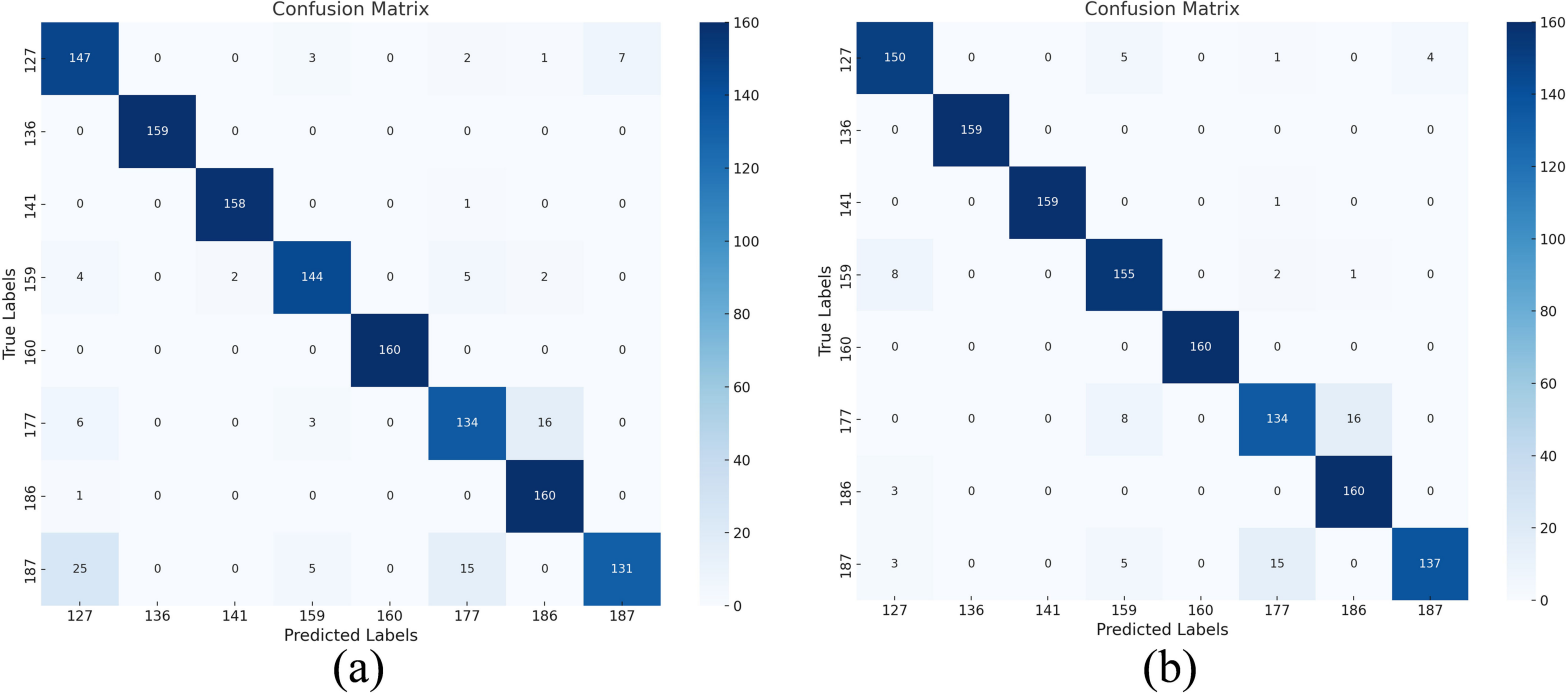
Comparison of introducing depth-separable convolution. Confusion matrix: **(a)** RGB&HSI-CBAM-ResNet50, **(b)** RGB&HSI-CBAM-ResNet50-DSC.

The confusion matrix before and after the implementation of DSC is contrasted in **Figure 15**. It is evident from this confusion matrix that the model can correctly identify samples of every category because the diagonal in **Figure 15b** has the great majority of correct classifications. Further evidence is that the CBAM-ResNet50-DSC model has greatly improved in feature extraction, and the critical area of emphasis is the low misclassifications in non-diagonal locations. Furthermore, the confusion matrix’s balanced classification performance for many categories demonstrates the model’s excellent generalization and stability, which offers a solid foundation for further applications.

The model maintains effective feature extraction capabilities while drastically lowering the computational cost thanks to deep separable convolution. Higher accuracy and robustness in the classification job are achieved by the model’s increased attention to the important feature regions when combined with the channel and spatial attention mechanism of CBAM. The design considerably improves the application value of multi-modal data fusion techniques and offers a superior solution for the sorghum seed classification job.

### 3.4 Results

**Table 4** shows that, compared to the previous model, the modified model improved several metrics for each of the eight sorghum seed types. Accuracy increased by 8.75%, 0.63%, 3.13%, 30.63%, 3.12%, 5.00%, 8.75%, and 5.62% for every sorghum seed. The corresponding improvements in recall were 8.75%, 0.62%, 3.12%, 27.12%, 3.13%, 5.00%, 6.91%, and 5.62%. The corresponding improvements in Precision were 10.02%, 5.39%, 11.49%, 12.79%, 3.13%, 12.13%, 0.10%, and 14.06%. Furthermore, there was a 0.0941, 0.0305, 0.0747, 0.2031, 0.0313, 0.0856, 0.0013, and 0.0963 improvement in the F1 scores, respectively. These findings suggest that the enhanced network performs better in recognition when categorizing maize seeds’ photos.

**Table 4:** Performance comparison of single-species models.

Seed category	Accuracy		Recall		Precision		F1-score	
	Before	After	Before	After	Before	After	Before	After
127	0.8500	0.9375	0.8500	0.9375	0.8144	0.9146	0.8318	0.9259
136	0.9875	0.9938	0.9875	0.9937	0.9461	1.0000	0.9664	0.9969
141	0.9625	0.9938	0.9625	0.9937	0.8851	1.0000	0.9222	0.9969
159	0.6625	0.9688	0.6625	0.9337	0.7681	0.8960	0.7114	0.9145
160	0.9688	1.0000	0.9687	1.0000	0.9687	1.0000	0.9687	1.0000
177	0.7875	0.8375	0.7875	0.8375	0.7545	0.8758	0.7706	0.8562
186	0.9125	1.0000	0.9125	0.9816	0.9799	0.9800	0.9450	0.9463
187	0.8000	0.8562	0.8000	0.8562	0.8108	0.9514	0.8050	0.9013

**Figure 16:**
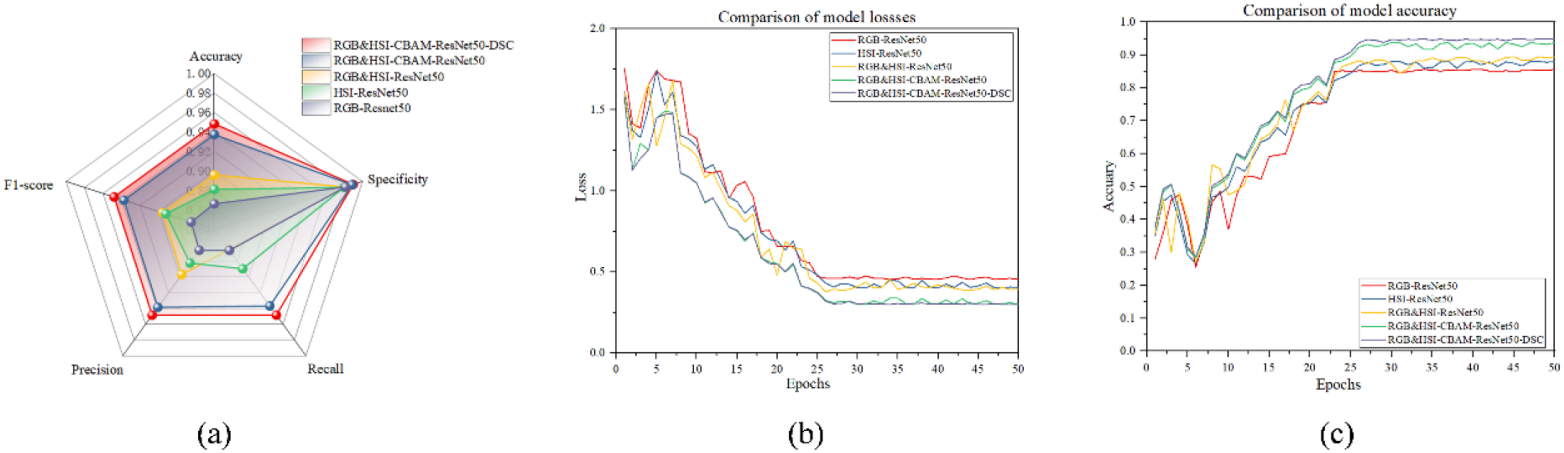
**(a)** Radar chart **(b)** Comparison of model losses **(c)** Comparison of model accuracy.

As can be observed from the radargram in **Figure 16a**, the RGB&HSI-CBAM-ResNet50-DSC model has the largest overall encompassing area and the strongest sorghum seed recognition ability. It also achieves the best results in all five performance metrics: Accuracy, Specificity, Recall, Precision, and F1-score. The RGB&HSI-CBAM-ResNet50 model, on the other hand, came in second, suggesting that the DSC module’s addition improved the model’s performance even further. The RGB&HSI-ResNet50 model outperformed the RGB-ResNet50 or HSI-ResNet50 models individually, suggesting that adding the attention mechanism and combining multi-modal information enhanced the model recognition effect. Regarding metrics like recall and F1-score, RGB-ResNet50 performs the lowest among them, indicating its limitations when tackling hyperspectral fine classification jobs. Therefore, multi-modal fusion and model optimization are crucial for improving model robustness and recognition.

The validation data loss for each epoch is displayed in **Figure 16b**. The loss value comparison graphs show that all five models’ loss values gradually decrease as the number of training rounds increases. This indicates that the models gradually converge during the training process. In the model performance comparison experiments, we compared the accuracy and loss values of the five models with the number of training rounds. With the lowest loss value, the RGB&HSI-CBAM-ResNet50-DSC model exhibits superior optimization and more effective training.

The accuracy of the validation data for every epoch is displayed in **Figure 16c**. The RGB&HSI-CBAM-ResNet50-DSC model attains the highest accuracy of over 90% at the late stage of training. Still, the accuracy of all the models rises with the number of training rounds, as seen in the accuracy comparison graph. This finding implies that the model’s classification performance can be considerably enhanced by integrating RGB and HSI information and adding CBAM and DSC methods. It suggests that enhanced network structure and greater feature information are necessary for the model to perform well on the job.

In conclusion, the findings demonstrate that the RGB&HSI-CBAM-ResNet50-DSC model achieves the best accuracy and loss values, confirming the usefulness of enhancing the model’s structure and combining multi-modal features.

To comprehensively evaluate the improvement in model performance and its statistical robustness, each model in this study was independently trained and tested ten times to ensure result stability and reproducibility. Based on these repeated experiments, a systematic statistical analysis was conducted using 95% confidence intervals and one-way analysis of variance (ANOVA) to assess classification performance across different models. The results indicate that with the gradual integration of hyperspectral information, multi-modal fusion strategies, the attention mechanism (CBAM), and depthwise separable convolution (DSC), the model accuracy steadily increased from 0.86596 for the baseline RGB-ResNet50 model (95% CI: [0.86513, 0.86679]) to 0.94779 for the final RGB&HSI-CBAM-ResNet50-DSC model (95% CI: [0.94727, 0.94831]). Furthermore, the narrowing of the confidence intervals indicates not only higher accuracy but also improved performance stability.

**Figure 17:**
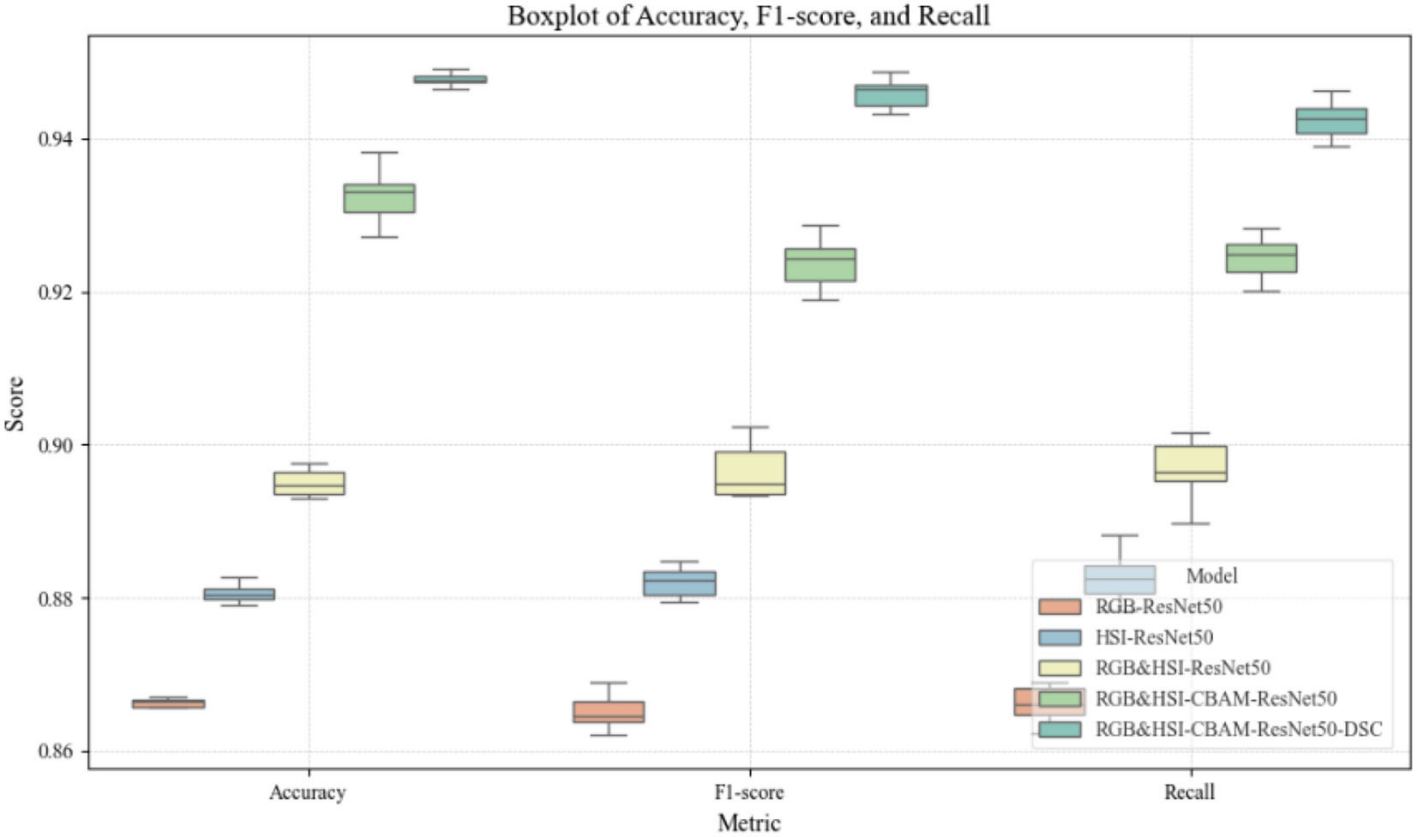
Boxplots of accuracy, F1-score, and recall for different models.

The subsequent ANOVA analysis revealed that the performance differences among the models were statistically highly significant (with p-values far below 0.05) across all five core evaluation metrics: Accuracy, F1-score, Recall, Precision, and Specificity. Additionally, three key indicators—Accuracy, F1-score, and Recall—were visualized using boxplots, as shown in **Figure 17**. These plots intuitively illustrate the median values and interquartile ranges of each model’s performance on different metrics. The results further confirm the trend of consistent performance improvement as the model architecture is gradually optimized. The final model demonstrated the best and most stable performance across all metrics. In summary, the combined statistical analysis and visualizations validate the effectiveness, robustness, and practical value of the proposed multi-modal fusion and structural optimization strategies in the classification task of sorghum seeds.

### 3.5 Ablation experiments

Using ResNet50 as the backbone, we conducted a series of ablation experiments to assess the impact of spectral fusion, attention mechanisms, and depthwise separable convolution (DSC) on model performance. The incorporation of the Convolutional Block Attention Module (CBAM) significantly improved classification accuracy by enhancing the model’s focus on critical spatial and channel-wise features. However, this enhancement came at the cost of increased parameter count and computational complexity, resulting in a slight rise in inference time. To mitigate this, DSC was introduced to replace standard convolutions, substantially reducing both the model size and computational load. Specifically, the number of parameters was reduced from 26.9 million to 17.4 million, while FLOPs decreased from 4.31G to 2.62G. When CBAM and DSC were combined, the model achieved the best trade-off between performance and efficiency-reaching the highest classification accuracy with only 18.3 million parameters and 2.92G FLOPs. Remarkably, the inference time remained comparable to or even slightly lower than the baseline model, highlighting the excellent potential of the proposed architecture for deployment in resource-constrained agricultural environments, as shown in **Table 5**.

**Table 5:** Comparison of ResNet50 experimental models with different module combinations.

ResNet50	Fusion	cbam	dsc	Accuracy	Params (M)	FLOPs (G)	Time (s)
✓				0.8664	25.6	4.10	0.0024
✓	✓			0.8961	25.6	4.10	0.0024
✓		✓		0.9107	26.9	4.31	0.0027
✓			✓	0.9024	17.4	2.62	0.0020
✓	✓	✓		0.9375	26.9	4.31	0.0027
✓	✓		✓	0.9389	17.4	2.62	0.0020
✓		✓	✓	0.9196	18.3	2.92	0.0023
✓	✓	✓	✓	0.9484	18.3	2.92	0.0023

✓ Indicates the presence of the corresponding module in the model. This setup is used to facilitate comparative analysis in the ablation study.

### 3.6 Validation of model generalization ability

To further assess the generalization ability and robustness of the proposed model, three publicly available seed image datasets from the Kaggle platform were selected for validation. As most public datasets contain only RGB images and lack corresponding hyperspectral data, only RGB-based evaluation was conducted in this section. The selected datasets include seven rice seed varieties, three maize seed varieties, and five soybean seed varieties. For each dataset, the performance of the proposed CBAM-ResNet50-DSC model was compared with two widely used baseline models, VGG16 and DenseNet121.As shown in **Table 6**, the proposed CBAM-ResNet50-DSC consistently achieved the highest classification accuracy across all three datasets-89.62% for rice seeds, 89.69% for maize seeds, and 94.73% for soybean seeds-outperforming both VGG16 and DenseNet121. These results demonstrate that the proposed model not only exhibits strong robustness and cross-dataset generalization but also adapts well to different categories of seed samples. Furthermore, the model shows broad applicability and practical value for real-world scenarios involving heterogeneous RGB image data from diverse sources.

**Table 6:** Sources and classification accuracy of different public seed datasets.

Data Source Link	Model	Accuracy
https://data.mendeley.com/datasets/v6vzvfszj6	VGG16	0.8452
	DenseNet121	0.8562
	CBAM-ResNet50-DSC	0.8962
https://doi.org/10.34740/kaggle/dsv/8681789	VGG16	0.8576
	DenseNet121	0.8745
	CBAM-ResNet50-DSC	0.8969
https://doi.org/10.34740/kaggle/dsv/6457847	VGG16	0.8838
	DenseNet121	0.9174
	CBAM-ResNet50-DSC	0.9473

## 4 Conclusions

This study proposes a sorghum seed variety classification approach based on RGB and hyperspectral (HSI) data fusion and an enhanced deep residual convolutional network (ResNet) for quick, nondestructive, and highly accurate seed identification. ResNet50 was utilized as the base network for model optimization by combining image data fusion with spectral data, the attention-based mechanism (CBAM), and the deep separable convolution (DSC). The spectral fusion dataset included 12,800 seeds from eight different varieties of sorghum seeds.

The experiment’s findings indicate that:

1. Multi-modal data fusion (RGB&HSI) can improve the model’s classification performance. The classification accuracy of the fused data is increased to 89.61%, and the F1-score is improved to 0.8959 compared with single data.
2. The model’s capacity to collect important features is further improved by adding the CBAM attention mechanism, raising the classification accuracy to 93.75%—4.14% higher than the base ResNet50.
3. The model’s computational efficiency is further maximized by combining it with depth separable convolution (DSC). In addition to lowering the number of model parameters and computational complexity, introducing DSC based on the CBAM-ResNet50 structure improves the final classification accuracy to 94.84%, a 1.09% improvement over the CBAM-ResNet50 model. This confirms the efficacy of the lightweight design.

Data fusion, attention mechanism optimization, and network lightweight design were used in this study to build an accurate and efficient sorghum seed classification model successfully. This model has high agricultural application value and offers a scientific foundation for variety screening and seed quality detection.

Although the proposed CBAM-ResNet50-DSC model based on 2D spectrogram fusion demonstrates excellent classification accuracy and robustness under experimental conditions, several challenges remain for real-world agricultural applications. First, the quality of RGB and hyperspectral images collected in field environments may be significantly affected by uncontrolled factors such as lighting variations, seed placement angles, image focus, and surface contamination (e.g., dust or aging). These factors can lead to instability in model predictions, thereby limiting practical effectiveness. Second, although this study simplifies the multi-modal fusion process at the model level, acquiring both RGB and hyperspectral images in practice still requires additional imaging equipment. This introduces extra hardware costs and operational complexity, which may hinder large-scale deployment in real field scenarios.

To address these issues, future research will focus on robustness enhancement strategies, domain adaptation techniques, and more cost-effective imaging solutions (e.g., compact multispectral sensors). Furthermore, incorporating multi-temporal or multi-angle data may further improve feature consistency and model stability, thus promoting the practical application of this method in precision agriculture.

## Data Availability

The raw data supporting the conclusions of this article will be made available by the authors, without undue reservation.
